# Nanometric alternating magnetic field generator

**DOI:** 10.1038/s41598-017-05026-4

**Published:** 2017-07-05

**Authors:** A. P. Espejo, F. Tejo, N. Vidal-Silva, J. Escrig

**Affiliations:** 1 Center for the Development of Nanoscience and Nanotechnology (CEDENNA), 9170124 Santiago, Chile; 2 Departamento de Fsica, Universidad de Santiago de Chile (USACH), Avda. Ecuador 3493, 9170124 Santiago, Chile

## Abstract

In this work we introduce an alternating magnetic field generator in a cylindrical nanostructure. This field appears due to the rotation of a magnetic domain wall located at some position, generating a magnetic region that varies its direction of magnetization alternately, thus inducing an alternating magnetic flux in its vicinity. This phenomenon occurs due to the competition between a spin-polarized current and a magnetic field, which allows to control both the angular velocity and the pinning position of the domain wall. As proof of concept, we study the particular case of a diameter-modulated nanowire with a spin-polarized current along its axis and the demagnetizing field produced by its modulation. This inhomogeneous field allows one to control the angular velocity of the domain wall as a function of its position along the nanowire allowing frequencies in the GHz range to be achieved. This generator could be used in telecommunications for devices in the range of radiofrequencies or, following Faraday’s induction law, could also induce an electromotive force and be used as a movable alternate voltage source in future nanodevices.

## Introduction

Magnetic domain walls (DWs) represent the local deformations of magnetization at the boundary of separation of two opposing and uniform magnetic domains. They are often present in the reversal processes of ferromagnetic nanostructures, varying their shape, size and behavior depending on the material and geometry of the nanostructure^1–6^. When a spin-polarized current passes through a magnetic material, the angular momentum of the spin of the electrons is transferred from the current to the local magnetization of the system, inducing a spin transfer torque (STT)^7^. It has been reported in many theoretical^8–10^ and experimental^11–14^ studies that this coupling allows one to control the movement of magnetic DWs by the application of a spin-polarized current^15^. The movement of DWs in ferromagnetic nanostructures opens a window of opportunities for potential applications in solid state systems and logic devices^16–19^.

The ability to move DWs using pulses of spin-polarized current has been studied mainly in planar geometries^12–14, 17^. The idea is to use this mechanism to control the information stored in a race-track memory^19^, information that is encoded in a magnetic nanowire through magnetic domains (up and down) separated by their DWs, respectively. In addition, it has been shown that the rotation of a DW can be induced by injecting a DC current into a magnetic wire with a DW. Using this simple concept, Ono *et al*.^20^ have proposed a novel three-terminal device that produces microwaves by utilizing the current-induced DW rotation. Other studies have investigated the DW oscillations upon the injection of a DC current through a geometrically constrained wire with perpendicular magnetic anisotropy^21–23^. All previously investigated systems may be useful as nanoscale microwave generators with possible applications in telecommunications or for rf-assisted writing in magnetic hard drives. From a fundamental point of view, the dynamics of DWs in planar systems is interesting due to certain complex features that are observed. For instance, when the DW is under the Walker breakdown limit^24, 25^, its velocity increases linearly with the field or current, and when the DW is above the limit, its structure changes periodically, causing a drastic decrease in its velocity.

**Figure 1:**
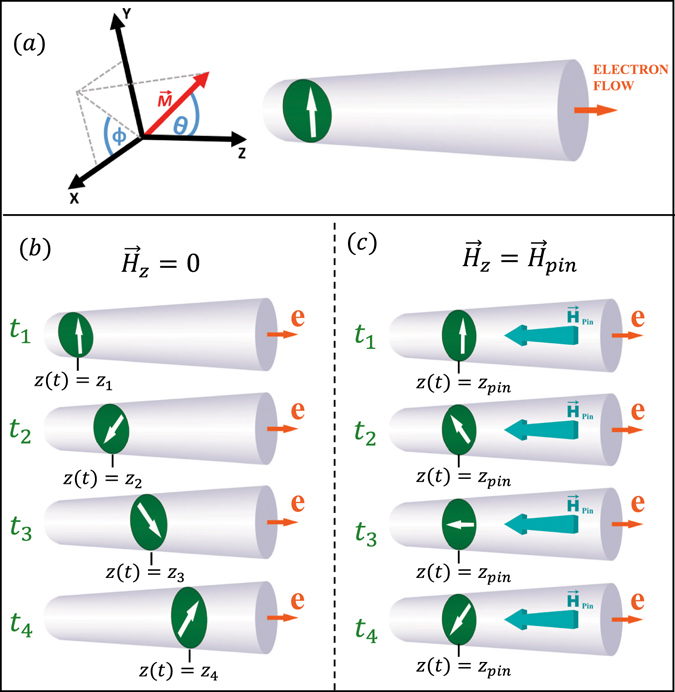
(**a**) The cartesian coordinate system and the spherical coordinate system used for the study of the TDW dynamics. The cross-sections indicate the position of the wall plane and the white arrows represent the orientation of the transverse magnetization. (**b**) The TDW moves linearly through the nanowire in the direction of electron flow (denoted with letter *e*) with a linear velocity proportional to $\dot{\theta }$ and rotates around the axis of symmetry with velocity $\dot{\varphi }$ in the absence of a magnetic field. (**c**) In the presence of a magnetic field ${H}_{z}={H}_{z}^{pin}$, the TDW stops its linear movement, however it keeps rotating with an angular velocity ${\dot{\varphi }}_{pin}$ in an equilibrium position *z*_*pin*_.

With the development of more sophisticated instruments and methods to synthesize and characterize nanostructures with complex geometries, the study of the current-driven DW motion has been extended to unconventional geometries. Recently, numerical simulations on the motion of DWs in cylindrical nanostructures have revealed that the dynamics of these DWs is very different from that observed in magnetic strips, specifically they showed that the Walker breakdown occurs at very high current densities^26^, or even can be suppressed^27^, thus introducing an advantage for the cylindrical systems in comparison with the magnetic strips. In these cylindrical systems, when a transverse domain wall (TDW) is under the influence of a magnetic field or a spin-polarized current, the wall acquires a translational motion, propagating in the same direction as the electron flow or applied field, and a rotation in the plane perpendicular to the main axis of the nanostructure, which periodically varies the magnetization of the nanostructure, generating an alternating magnetic field^27^. Both motions occur simultaneously while the nanostructure continues under the influence of a magnetic field or current (see Fig. 1b).

Considering the work previously mentioned, in this work we present a time-stable nanometric alternating magnetic field generator, which is capable of maintaining the rotation of the DW in a fixed position, thus permitting the *in*-*situ* knowledge of the source of field variation. In this way, we will establish the relation that must be satisfied by the spin-polarized currents and the magnetic fields to obtain the phenomenon described previously. As proof of concept, we will show that by means of a spin-polarized current and a demagnetizing field, created by the geometric change of a modulated diameter nanowire, it is possible to fix the position of a TDW, and generate a stable rotation of this DW.

## Analytical Model

Initially, we will consider a nanowire with cylindrical symmetry containing a TDW. We will also consider that the DW is simultaneously under the action of a magnetic field and a spin-polarized current. In this way, the dynamics of the TDW can be estimated by the Landau-Liftshitz-Gilbert equation, with the inclusion of additional STT terms^28, 29^:

$$\frac{d\vec{M}}{dt}=\gamma {\vec{H}}_{eff}\times \vec{M}+\frac{\alpha }{{M}_{s}}[\vec{M}\times \frac{d\vec{M}}{dt}]-(\vec{u}\cdot \vec{\nabla })\vec{M}+\frac{\beta }{{M}_{s}}\vec{M}\times [(\vec{u}\cdot \vec{\nabla })\vec{M}]$$

where $\vec{M}$ is the local magnetization, ${\vec{H}}_{eff}$ the effective field, *M*_*s*_ the saturation magnetization, *γ* the gyromagnetic ratio, *α* the Gilbert damping factor, and *β* the non-adiabatic spin-transfer parameter. The vector $\vec{u}$ is defined as $\vec{u}=-(g{\mu }_{B}P\mathrm{/2}e{M}_{s})\vec{J}$, where *g* is the Landé factor, *μ*_*B*_ the Bohr magneton, *e* the electron charge, $\vec{J}$ the current density and *P* the polarization rate of the current (*u* is positive for *P* > 0). For simplicity, we will use $\vec{u}=\rho \vec{J}$, where *ρ* is a constant of proportionality.

As a starting point, we will use the equations proposed by Yan *et al*.^27^, who adapted the analytic model proposed by Mougin *et al*.^25^. These equations describe the velocity of a single 180° DW in a magnetic nanostructure of reduced dimensions with cylindrical symmetry under the action of an external applied magnetic field and/or an electrical current. The linear velocity of the DW is proportional to $\dot{\theta }$, while the angular velocity of rotation is $\dot{\varphi }$. When the TDW is driven just by a static field applied along the *z* direction (*H* = *H*_*z*_, *u* = 0), the velocities are given by $\dot{\theta }=-\alpha \dot{\varphi }$ and $\dot{\varphi }=\alpha {H}_{z}\mathrm{/(1}+{\alpha }^{2})$, and the velocities of the magnetization due to the current-driven case (*H*_*z*_ = 0, *u* = *u*_*z*_) are given by $\dot{\theta }={-[(1+\alpha \beta ){u}_{z}\mathrm{/(1}+{\alpha }^{2})]{\partial }_{z}\theta (z)|}_{wc}$ and $\dot{\varphi }={[(\beta -\alpha ){u}_{z}\mathrm{/(1}+{\alpha }^{2})]{\partial }_{z}\theta (z)|}_{wc}$, where *wc* denotes the center of the TDW (*θ* = *π*/2), *θ*(*z*) is the profile of the wall and *u*_*z*_ is the vector in the *z*-direction defined previously. Following from these ideas, we explicitly write the equations that consider the combined action of an external magnetic field and a spin-polarized current,

1 $$\dot{\theta }=\frac{\gamma \alpha {H}_{z}}{(1+{\alpha }^{2})}+\frac{(1+\alpha \beta )\,\rho {J}_{z}}{(1+{\alpha }^{2})\,{\rm{\Delta }}}$$

2 $$\dot{\varphi }=-\frac{\gamma {H}_{z}}{(1+{\alpha }^{2})}-\frac{(\beta -\alpha )\,\rho {J}_{z}}{(1+{\alpha }^{2})\,{\rm{\Delta }}}$$

In these equations, Δ corresponds to the characteristic wall width, while *u*_*z*_ = *ρJ*_*z*_. From the above equations it can be deduced that a TDW can remain rotating in a fixed position if the condition of $\dot{\theta }=0$ occurs. Then, to stop the propagation of the TDW, it is required that the magnetic field and the electron flow have opposite directions, satisfying the following relation (see Fig. 1c):

3 $${H}_{z}^{pin}({J}_{z})=-\frac{(1+\alpha \beta )}{\alpha \gamma {\rm{\Delta }}}\rho {J}_{z}.$$

Under these circumstances, combining Eqs (2) and (3), we obtain the angular velocity, ${\dot{\varphi }}_{pin}({J}_{z})$, when the TDW remains pinned,

4 $${\dot{\varphi }}_{pin}({J}_{z})=\frac{(1+\alpha \beta -\beta +\alpha )}{\alpha (1+{\alpha }^{2})\,{\rm{\Delta }}}\rho {J}_{z}.$$

**Figure 2:**
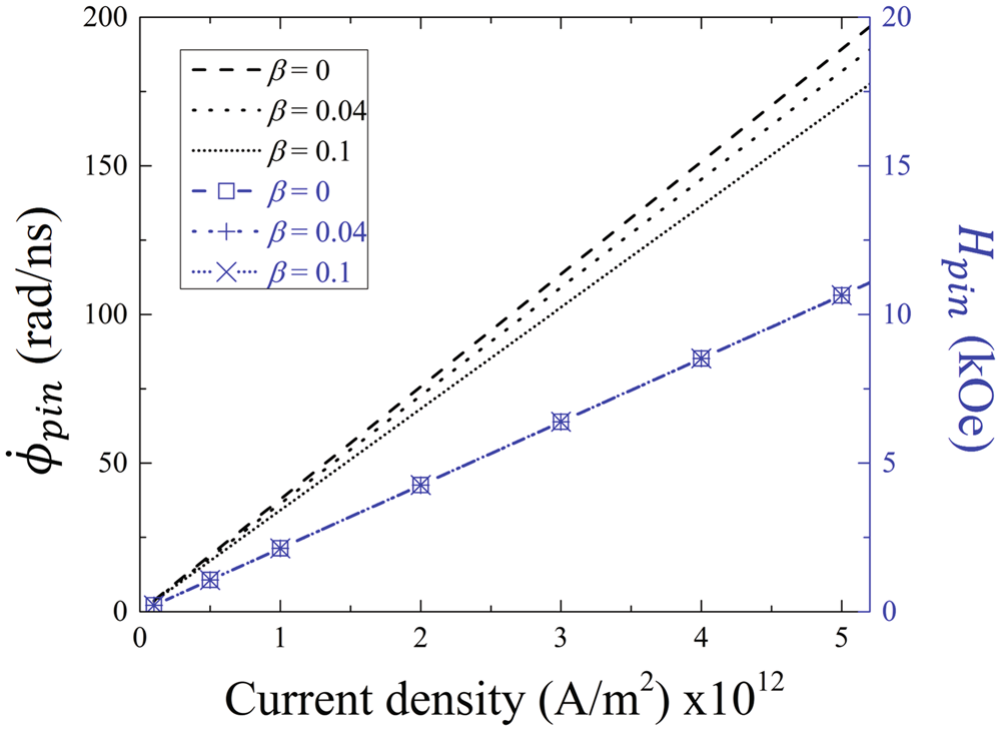
Estimation of the angular velocity ${\dot{\varphi }}_{pin}$ (obtained from Eq. 4, black lines) and magnetic field ${H}_{z}^{pin}$ (obtained from Eq. 3, blue lines and symbols) of the DW for different current densities.

Figure 2 shows the magnetic field ${H}_{z}^{pin}$ (obtained from equation 3) and the angular velocity ${\dot{\varphi }}_{pin}$ (obtained from equation 4) as a function of the current density, with values typically used in experiments. From this figure we can observe both the magnetic field and the angular velocity of rotation increase linearly with the current density. In addition, it is interesting to note that this angular velocity of the pinned TDW reaches GHz, which allows this configuration to have applications in the area of communications.

## Micromagnetic Simulations

**Figure 3:**
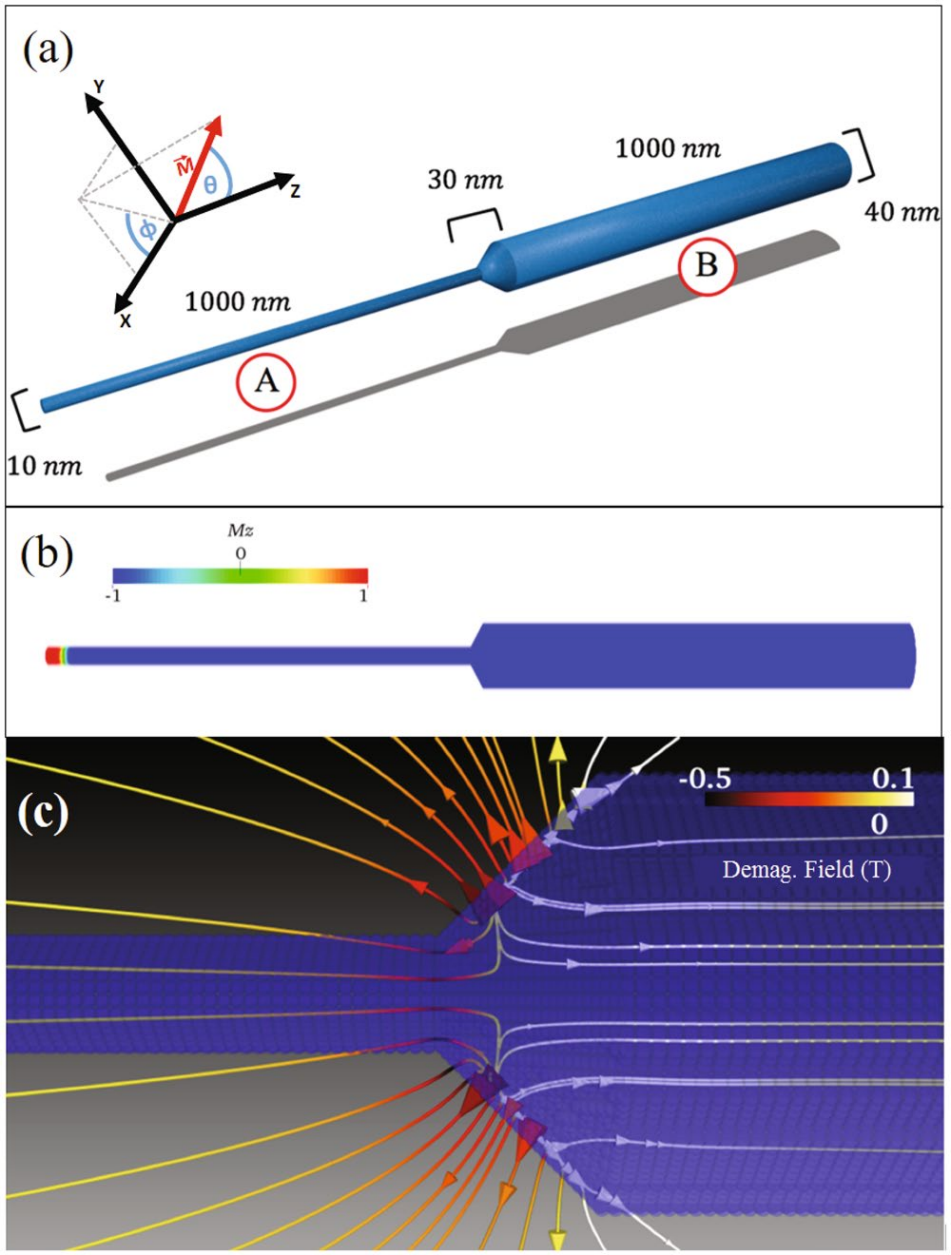
(**a**) Geometrical characterization of the simulated nanowire with modulated diameter. (**b**) Initial state of the system: a TDW moves due to the application of a electron flow in +*z* direction. (**c**) Demagnetizing field lines in the transition zone of the modulated nanowire.

As proof of concept, we will consider a demagnetizing field created by the geometric change of a cylindrical nanowire with modulated diameter, by means of which it is possible to fix the position of a TDW, and this position generates a rotation of the DW stable in time. For this, we performed numerical simulations using a micromagnetic simulator^30^ extended to consider also the dynamics of the magnetization induced by a spin-polarized current^12^. Our starting point is a cylindrical diameter modulated *Ni*_80_ *Fe*_20_ nanowire with a total length *L* = 2030 nm. The nanowire is composed of three segments: the segment A has a diameter *d*_*A*_ = 10 nm and a length *L*_*A*_ = 1000 nm, the segment B has a diameter *d*_*B*_ = 40 nm and a length *L*_*B*_ = 1000 nm, and between both segments, the diameter decreases linearly from 40 to 10 nm over a 30 nm length, as shown in Fig. 3a.

We have used a saturation magnetization of *M*_*S*_ = 800 × 10^3^ A/m, a stiffness constant *A* = 13 × 10^−12^ J/m and cell sizes of 1.25 × 1.25 × 2.5 nm^3^. Besides we have neglected the crystalline anisotropies since the nanowires are polycrystalline. Additionally, the current density varies with the transverse area of the nanostructure and, from now on, when we talk about simulations with electric current, we will refer to the current density of segment A. Finally, we use *α* = 0.01, *β* = 0.04, and *P* = 0.4^26, 29^.

**Figure 4:**
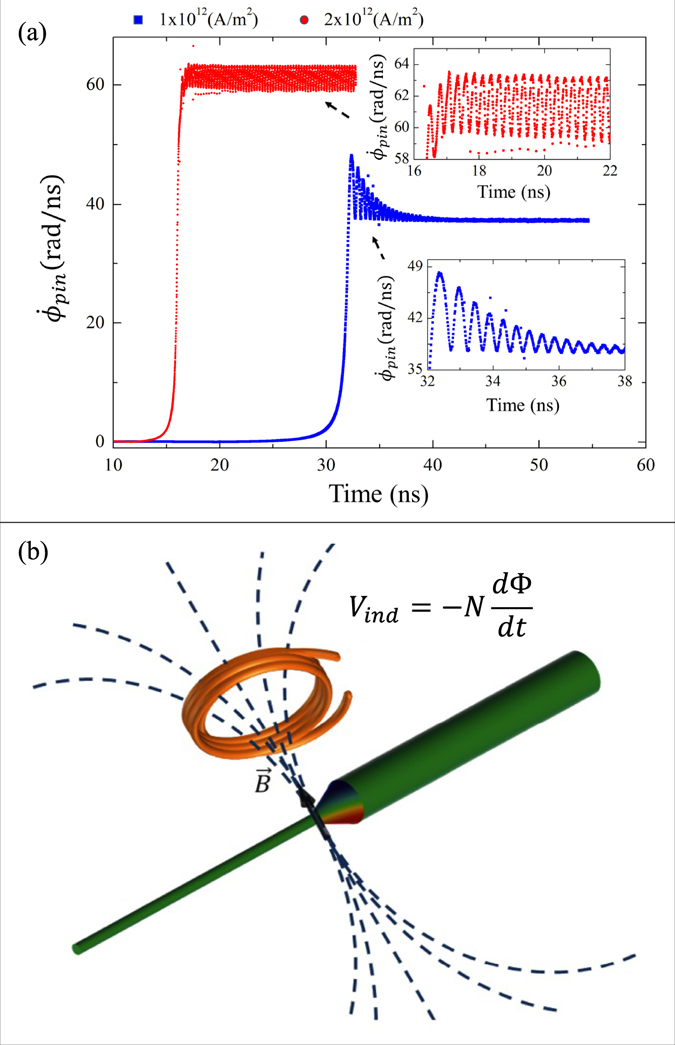
(**a**) Angular velocity for two current densities, where the TDW finds an equilibrium position due to competition between the electric current and the demagnetizing field. (**b**) Scheme of a nanogenerator where the generated alternating magnetic field induces an alternating voltage in a neighboring circuit.

Figure 3b shows the initial configuration of the system, where a TDW lies near the free end of segment A, and due to this it minimizes energy versus other types of DWs^3^. Then, at *t* = 0 s, an electric current is applied with current densities *J* ranging between 1 × 10^12^ A/m^2^ and 1 × 10^13^ A/m^2^, as typically used in experiments^19, 31–33^. It is important to consider that these high current densities can only be applied in pulses which typically last less than 100 ns. In our case, and as we will show later (see Fig. 4a), the angular velocity will generally converge for times less than 40 ns, so that if a 100 ns pulse is applied, the system will exhibit a constant angular velocity for about 60 ns. The current densities used in our simulations produce an Oersted field that can be neglected^29^, and has therefore not been considered in our simulations. An electric current along −*z* displaces the TDW towards the +*z* direction, i.e., in the electron flow direction (from segment A to segment B).

The advantage of using a diameter-modulated nanowire is to replace the source of the external magnetic field by the demagnetizing field created naturally by the modulation and segment *B*. As can be seen in Fig. 3c, the demagnetizing field *H*_*d*_ in the thin nanowire region is inhomogeneous and it is oriented in the −*z* direction, which could allow one to slow the propagation of the TDW with direction +*z*, driven by an electric current. Due to the non-homogeneity of the demagnetizing field along the wire for certain values of current density, it is possible to find an equilibrium position *z*_*cr*_ satisfying Eq. (3) but not suppressing its rotation, where ${H}_{d}({z}_{cr})={H}_{z}^{pin}$. From our simulations, we can obtain that at *z* = 1000 nm, the demagnetizing field *H*_*d*_ reaches values close to 0.5 T in the *−*z direction, and decreases rapidly along the segment A. Thus, when the TDW is far from the modulation, the effect of the demagnetizing field on the DW is negligible. However, when the TDW is near the interface, the influence of the demagnetizing field becomes important and both $\dot{\theta }$ and $\dot{\varphi }$ velocities are affected.

From the results obtained one may observe a linear relationship between the speed of propagation of the TDW in the segment A and the current density. Once the TDW approaches and reaches the modulation, the DW strongly slows its propagation, increasing its angular velocity and two possible results are generated. First, if the current is sufficiently high, *J* > 2 × 10^12^ A/m^2^, the TDW will continue its movement towards and along the segment B, significantly reducing its velocity, which will be proportional to the decrease in current density due to the change in diameter from *d*_*A*_ = 10 nm to *d*_*B*_ = 40 nm. On the other hand, for low current densities, *J* ≤ 2 × 10^12^ A/m, the TDW will stop its displacement at a point near the modulation due to the contribution of the demagnetizing field when it is equal to that of the spin-polarized current, thus finding the equilibrium position *z*_*cr*_ satisfying Eq. (3).

Figure 4a shows the behavior of the angular velocity ${\dot{\varphi }}_{pin}$ of the TDW as a function of time for the regime of a low current density. We find that for *J* = 1 × 10^12^ A/m^2^, the TDW stops its motion at *z*_*cr*_ = 996.5 nm, but it continues rotating to reach a maximum angular velocity of 48.2 rad/ns. Then, the angular velocity varies periodically over time until it converges to a stable value of 37.2 rad/ns, which is equivalent to a frequency of 5.92 GHz, in the microwave range. At this point it is important to mention that we have performed micromagnetic simulations for different values of the non-adiabatic spin-transfer parameter *β* (not shown here), and the frequency variation is less than 3%. From Eq. (4), and considering a current density of *J* = 1 × 10^12^ A/m^2^, we have obtained an angular velocity ${\dot{\varphi }}_{pin}=36.6\,{\rm{rad}}/{\rm{ns}}$ that is in good agreement with the value obtained from the micromagnetic simulations.

Moreover, we find that for *J* = 2 × 10^12^ A/m^2^ the TDW stops its motion into the modulation at *z*_*cr*_ = 1002 nm, but it continues rotating to reach an average angular velocity of 61 rad/ns that is equivalent to a frequency of 9.78 GHz, with periodic variations between 59 rad/ns and 63 rad/ns. This frequency variation is due to the overlap between the TDW and the spin waves generated when the TDW enters the modulated zone. For higher currents densities a more intense magnetic field is necessary to stop the TDW. However, the modulation zone generates a magnetic field whose maximum value is −0.5 T, which is not enough to stop the TDW when it moves due to the application of current densities *J* > 2 × 10^12^ A/m^2^.

The behavior described above not only refers to the geometric parameters chosen for the simulated nanostructure, but it will in principle work for any nanostructure that satisfies equation 4. In fact, if a conductive circuit is placed near the nanostructure, then an electromotive force will be induced because of the change in the magnetic flux created by the rotation of the TDW, as shown in Fig. 4b. In this way, we have proposed this nanostructure as a nanometric alternating magnetic field generator of controlled frequency and position^34^.

In the literature there are several studies that show that the width of the domain walls increases while the magnetization decreases with an increase in temperature^35–37^. The model used in this article considers that there is a magnetic field that stops the displacement of a domain wall. In this case, the magnetic field is assumed to be approximately constant within the domain wall. However, if the temperature increases considerably the domain wall width, probably the magnetic field will no longer be constant inside the wall. Furthermore, increasing the temperature decreases the magnetization of the nanowire, which implies that the demagnetizing field will decrease and probably will not be able to stop the domain wall.

On the other hand, there are numerous articles^38–42^ which indicate that a current density in the order of 10^12^ A/m^2^ generates a temperature of about 0–100 K for each ns of pulse over a nanowire. Of course, the temperature increase will depend on the geometric and magnetic parameters, the magnitude and time of the pulse, and the substrate where the nanowire will be supported. For all of the above, our model is valid for low temperatures, and therefore, in order to operate the device presented in this article, suitable conditions must be sought so that the applied current density does not considerably increase the temperature of the device. A good thermal conductor as a substrate is an alternative to achieve a lower average temperature. For example, Fangohr *et al*.^39^ showed that a Permalloy nanowire with notch placed on a silicon wafer exhibits a maximum temperature increase of 17 K when a 20 ns pulse (current density at the wire ends is 10^12^ A/m^2^) is applied, conditions very similar to those exhibited by our nanogenerator. In addition, Parkin *et al*.^19^ showed that by choosing a suitable substrate it is possible to move magnetic domain walls using pulses between 20 and 100 ns with a current density in the order of 10^12^ A/m^2^, same order of magnitude used in our investigation.

## Conclusions

In conclusion, we have demonstrated through analytical calculations and micromagnetic simulations that the competition between a spin-polarized current and a magnetic field allows to stop the translation movement of a TDW traveling along a cylindrical geometry, but without stopping its angular movement, generating a magnetic region that varies its direction of magnetization alternately, thus inducing an alternating magnetic flux. From a theoretical point of view, we have obtained analytical expressions for both the magnetic field ${H}_{z}^{pin}$ necessary to stop the translation movement of the DW (Eq. (3)) and its angular velocity ${\dot{\varphi }}_{pin}$ (Eq. (4)). Interestingly, both expressions increase linearly with the current density. Furthermore, as proof of concept, we have investigated the propagation of a TDW driven by a spin-polarized current in a cylindrical diameter modulated *Ni*_80_ *Fe*_20_ nanowire by means of micromagnetic simulations. It is observed that once the TDW approaches and reaches the region of change of diameter, the DW strongly decelerates its propagation, completely stopping its displacement for certain current densities. Finally, we obtained that the angular velocity of the pinned TDW is in the microwave range, which allows these structures to have applications in communications.
